# Developmental programming of obesity by maternal exposure to concentrated ambient PM_2.5_ is maternally transmitted into the third generation in a mouse model

**DOI:** 10.1186/s12989-019-0312-6

**Published:** 2019-07-02

**Authors:** Yanyi Xu, Wanjun Wang, Minjie Chen, Ji Zhou, Xingke Huang, Shimin Tao, Bin Pan, Zhouzhou Li, Xiaoyun Xie, Weihua Li, Haidong Kan, Zhekang Ying

**Affiliations:** 1 Department of Environmental Health, School of Public Health, Fudan University, 130 Dong’an Rd, Shanghai, 200032 China; 2 Shanghai Key Laboratory of Meteorology and Health, Shanghai Meteorological Service, Shanghai, China; 3 Department of Medicine Cardiology Division, University of Maryland School of Medicine, 20 Penn St. HSFII S005, Baltimore, MD 21201 USA; 4 NHC Key Laboratory of Reproduction Regulation (Shanghai Institute of Planned Parenthood Research), Fudan University, Shanghai, China; 5 Department of Interventional & Vascular Surgery, Shanghai Tenth People’s Hospital, Tongji University School of Medicine, Shanghai, China

**Keywords:** PM_2.5_, Maternal exposure, Obesity, Developmental programming, Cross-generational transmission

## Abstract

**Background:**

Obesity is an uncontrolled global epidemic and one of the leading global public health challenges. Maternal exposure to ambient fine particulate matter (PM_2.5_) may adversely program offspring’s adiposity, suggesting a specialized role of PM_2.5_ pollution in the global obesity epidemic. However, the vulnerable window for this adverse programming and how it is cross-generationally transmitted have not been determined. Therefore, in the present study, female C57Bl/6 J mice were exposed to filtered air (FA) or concentrated ambient PM_2.5_ (CAP) during different periods, and the development and adulthood adiposity of their four-generational offspring were assessed.

**Results:**

Our data show that the pre-conceptional but not gestational exposure to CAP was sufficient to cause male but not female offspring’s low birth weight, accelerated postnatal weight gain, and increased adulthood adiposity. These adverse developmental traits were transmitted into the F_2_ offspring born by the female but not male F_1_ offspring of CAP-exposed dams. In contrast, no adverse development was noted in the F_3_ offspring.

**Conclusions:**

The present study identified a pre-conceptional window for the adverse programming of adiposity by maternal exposure to PM_2.5_, and showed that it was maternally transmitted into the third generation. These data not only call special attention to the protection of women from exposure to PM_2.5_, but also may facilitate the development of intervention to prevent this adverse programming.

## Introduction

The World Health Organization (WHO) estimates that more than 90% of the current world population still live in places exceeding the WHO air quality guidelines (www.who.int/phe/ publications/air-pollution-global-assessment/en/). Ambient fine particulate matter (PM_2.5_) is one of the criterion air pollutants. A big number of studies have demonstrated that exposure to PM_2.5_ correlates with increased premature mortality and various cardiopulmonary diseases [1]. The biological mechanism for these associations, however, has not yet been fully understood. A focused scientific effort over the last decade demonstrates that parental exposure to environmental stressors may persistently impact offspring’s susceptibility to a variety of non-communicable diseases including obesity, referred to as the developmental origins (or programming) of health and disease (DOHaD) [2]. Notably, our studies and others’ strongly suggest that maternal exposure to PM_2.5_ may be a “programming” factor for obesity and persistently effect the offspring’s health. Specifically, numerous epidemiological studies have shown that maternal exposure to PM_2.5_ correlates with a variety of developmental perturbation, from low birth weight to abnormal pubertal development [3–13]. As per the DOHaD paradigm, early-life perturbation is a prerequisite for developmental programming [14]. Furthermore, several epidemiological studies have investigated the impact of maternal exposure to PM_2.5_ on offspring’s susceptibility to obesity, and almost all of them support that maternal exposure to PM_2.5_ is obesogenic [8, 15–19].

Toxicological studies also support that maternal exposure to PM_2.5_ programs offspring’s susceptibility to obesity. For example, in utero exposures to concentrated ambient PM_2.5_ (CAP) or diesel exhaust decreases the birth weight of newborn mice [20–26]. Maternal exposure to ambient pollutants increases body weight of adult offspring [20, 21] and aggravates their high fat diet-induced obesity [27]. Consistent with these toxicological studies, we previously demonstrated that maternal intratracheal instillation of diesel exhaust PM_2.5_ (DEP) altered offspring’s growth trajectory and increased their adulthood adiposity [28]. More importantly, we found that maternal exposure to CAP throughout a 7-week pre-conception period and the whole gestation and lactation period significantly reduced offspring’s birth weight and rendered male but not female offspring a marked increase in adulthood adiposity [29]. Intriguingly, although the gestation and lactation period is frequently identified as a vulnerable window for developmental programming by maternal exposure to environmental stressor [30, 31], maternal exposure to CAP throughout the gestation and lactation period only was insufficient to program offspring’s growth trajectory [29], warranting further study to determine the vulnerable window for this adverse programming. In addition, developmental programming has frequently been shown to be multiple-generationally transmissible, but whether the programming of offspring’s adiposity by maternal exposure to PM_2.5_ is multi-generationally transmissible is not yet investigated. Therefore, in the present study, female C57Bl/6 J mice were exposed to filtered air (FA) or CAP during different periods, and the development and adulthood adiposity of their four-generational offspring were assessed.

## Methods

### Whole-body inhalation exposure to CAP and breeding protocols

**Fig. 1:**
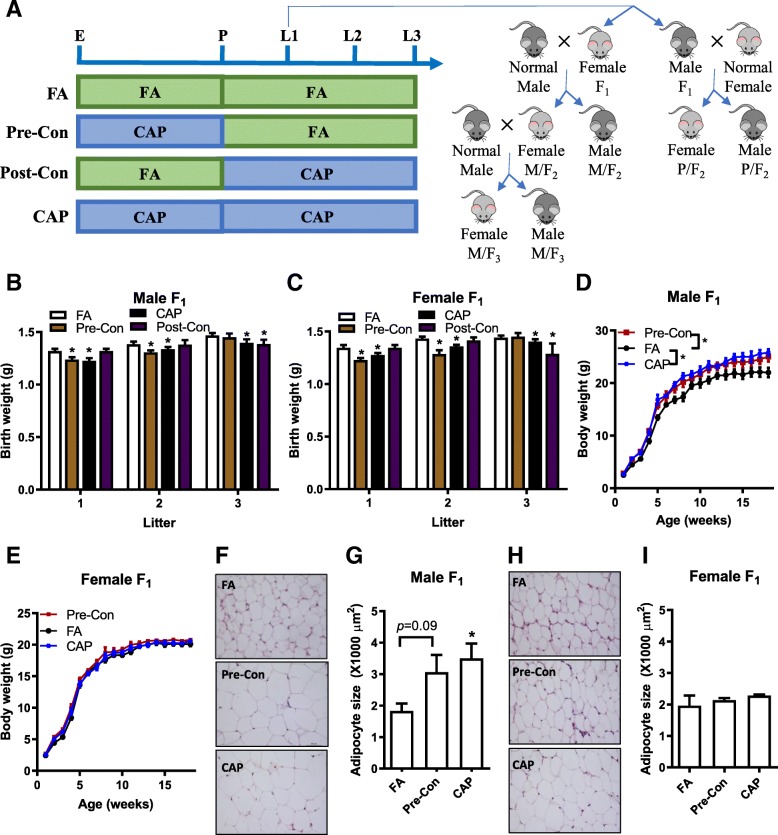
Maternal pre-conception exposure to CAP is sufficient to decrease offspring’s birth weight and increase adulthood adiposity. **a** Exposure scheme: E, start of exposure; P, presence of sperm plug; L1, birth of litter one; L2, birth of litter two; L3, birth of litter three. **b**. The birth weight of male F_1_ offspring. *n* = 20–33/group. **c** The birth weight of female F_1_ offspring. *n* = 9–35/group. **d** The growth trajectory of male F_1_ offspring. **e** The growth trajectory of female F_1_ offspring. **f** and **g**. The representative histological (H&E staining) images (**f**) and adipocyte size (**g**) of male F_1_ offspring’s perigonadal adipose tissues obtained using ImageJ. **h** and **i**. The representative histological (H&E staining) images (**h**) and adipocyte size (I) of female F_1_ offspring’s perigonadal adipose tissues obtained using ImageJ. *n* = 12–30/group, *p < 0.05 versus FA, one-way or two-way ANOVA

All the procedures of this study were approved by the Institutional Animal Care and Use Committee at Fudan University, and all the animals were treated humanely and with regard for alleviation of suffering. C57Bl/6 J mice (female, 3-week-old) were purchased from the Animal Center of Shanghai Medical School, Fudan University (Shanghai, China) and were housed in standard cages with a 12-h light/12-h dark cycle with temperatures of 18–25 °C and relative humidity of 40–60%. After one-week acclimation, mice were exposed to filtered air (FA, *n* = 20) or CAP (*n* = 20) using a versatile aerosol concentration enrichment system (VACES) that was modified for long-term whole-body exposures. The exposure protocol comprised exposures for 6 h/day and 5 days/week (no exposure took place during the weekend). After a 7-week exposure to FA/CAP, all the mice were mated with normal C57Bl/6 J mice (purchased from the Animal Center of Shanghai Medical School, Fudan University, 12-week-old, 1 male mating with 1 female). During the first week of mating, the presence of sperm plug was checked daily. Upon the presence of sperm plug, the dams were either maintained in the same exposure or switched to the other, alternately. As such, there were four groups of FA/CAP-exposed dams (F_0_, *n* = 10/group): 1) FA, exposed to FA only; 2) Post-Con, exposed to FA pre-conceptionally and exposed to CAP post-conceptionally; 3) CAP, exposed to CAP only; 4) Pre-Con, exposed to CAP pre-conceptionally and exposed to FA post-conceptionally (Fig. 1a). Since then, all the F_0_ dams were consecutively subjected to the same exposure until the birth of the litter 3 offspring (litters 2 and 3 pups were weighed and euthanized shortly after birth). The sires and pups were housed in standard cages and not exposed to FA/CAP throughout the whole period of experiment.

The F_1_ offspring (litter 1 only, as shown in Fig. 1a) were mated with normal male or female C57Bl/6 J (12-week-old, one male mating with one female) after recording the growth trajectory. Therefore, the F_2_ offspring in the present study were born either by the female F_1_ offspring of FA- or CAP-exposed dams (the maternal line, M/F_2_) or by the male F_1_ offspring of FA- or CAP-exposed dams (the paternal line, P/F_2_). Because developmental programming of adulthood adiposity was observed in M/F_2_ but not in P/F_2_, only the female M/F_2_ were mated with normal male C57Bl/6 J (12-week-old, one male mating with one female) to generate F_3_.

### Growth trajectory recording

To minimize the effect of litter size on the offspring growth trajectory, all pups were indiscriminately culled to 5–6/litter on the postnatal day 3 by a technician blind to the grouping. All pups were weaned at postnatal week 3, and then housed in standard cages with standard rodent diet. All the weanlings were housed 3–5 mice/cage, and weighed weekly until 18 weeks old.

### Body composition analysis

On the day of experiment, after measurement of their body weight and length, all the mice were euthanized and their blood was harvested from the orbital venous plexus. Heart, lung, liver, kidney, pancreas, spleen, testis/ovary, perirenal adipose tissue, epididymal/parametrial adipose tissue, subcutaneous adipose tissue, and brown adipose tissue (BAT) were weighted, fixed in 4% paraformaldehyde for morphological analysis and/or snap-frozen in liquid nitrogen and then stored at − 80 °C for further use.

### Histological analysis of adipose tissues

Epididymal adipose tissues were fixed in 4% paraformaldehyde, embedded in paraffin, cut into 5-μm sections, and subjected to hematoxylin & eosin staining. Images at 40× magnification were obtained with a SPOT digital camera (Diagnostic Instruments, Sterling Heights, MI) by a technician blind to the grouping. The cross-sectional areas of adipocytes in epididymal adipose tissues were calculated as previously described [32].

### Leptin mRNA expression analysis by qPCR

Total RNAs were extracted from the frozen epididymal adipose tissues, and the leptin mRNA expression levels were assessed by qPCR as previously described [29].

### Statistics

All data are expressed as means ± SEMs unless noted otherwise. Statistical tests were performed using one-way or two-way analysis of variance (ANOVA) followed by Bonferroni correction (either compare all pairs [Figs. 1d and e] or compare to FA [all other Figures]) or unpaired student’s *t* test using GraphPad Prism (version 5; GraphPad Software, La Jolla, CA, USA). The significance level was set at *p* < 0.05.

## Results

### Maternal pre-conception exposure to CAP is sufficient to decrease offspring’s birth weight

We previously demonstrated that maternal CAP exposure throughout a 7-week pre-conception period and the whole gestation and lactation period programs the offspring’s development and increases their adulthood adiposity [29]. To identify the vulnerable window for this adverse developmental programming by maternal PM_2.5_ exposure, female C57Bl/6 J mice were exposed to FA or CAP for 7 weeks, and then mated with normal male C57Bl/6 J mice. Upon the presence of sperm plug, the dams were either maintained in the same exposure or switched to the other. Therefore, there were four groups of FA/CAP-exposed dams (F_0_): 1) FA, exposed to FA only; 2) Post-Con, exposed to FA pre-conceptionally and exposed to CAP post-conceptionally; 3) CAP, exposed to CAP only; and 4) Pre-Con, exposed to CAP pre-conceptionally and exposed to FA post-conceptionally (Fig. 1a).

The ambient PM_2.5_ concentration and the average PM_2.5_ concentrations in the FA and CAP chambers are presented in Table 1. The compositions of ambient PM_2.5_ and CAP were previously reported, characterized by the relatively high crustal elements including Si, Al, Ti, and Fe [33] We did not observe any significant difference in gestation duration, litter size, and offspring sex ratio (Table 2).

**Table 1:** PM_2.5_ concentrations (mean ± SD) during different periods. ^of litter 1. ^%^including the week when checking the sperm plug

	Ambient (ug/m^3^)	FA (ug/m^3^)	CAP (ug/m^3^)
Preconception^^,%^ (56 days)	32.9 ± 21.8	12.1 ± 4.7	183.2 ± 92.9
Gestation^^,%^ (26 days)	43.3 ± 22.5	14.3 ± 7.4	217.7 ± 121.3
Lactation^ (24 days)	56.2 ± 34.3	16.9 ± 8.7	297.3 ± 127.5
The remaining observation period (185 days)	51.3 ± 37.4	16.5 ± 9.2	289.3 ± 139.4

**Table 2:** Characterization of litter 1 offspring. Data are presented as mean ± SD. *n* = 6–7/group

	FA	CAP	Pre-Con	Post-Con
Gestation (days)	20.8 ± 0.4	20.5 ± 0.3	20.5 ± 0.5	20.3 ± 0.4
Litter size	6.4 ± 2.1	6.3 ± 3.1	6.0 ± 2.6	6.5 ± 3.1
Sex ratio (male/female)	0.8 ± 0.7	1.1 ± 0.9	1.0 ± 0.9	0.8 ± 1.3

Figures 1b and c demonstrate that consistent with our previous study [29], maternal exposure to CAP throughout the 7-week pre-conceptional period and the whole gestational period (litter 1, CAP versus FA) significantly decreased the offspring’s birth weight. Notably, CAP exposure throughout the 7-week pre-conceptional period only also significantly decreased the offspring’s birth weight (Figs. 1b and c litter 1, Pre-Con versus FA), whereas CAP exposure throughout the gestational period only did not significantly affect offspring’s birth weight (Figs. 1b and c litter 1, Post-Con versus FA). Additionally, there was no significant difference between the birthweight of offspring born by the CAP dams and those born by the Pre-Con dams (Figs. 1b and c litter 1). These data strongly suggest that maternal pre-conceptional exposure to CAP is sufficient to program the offspring’s development.

To ascertain how long the developmental programming capacity would be maintained in those CAP-exposed dams, all the dams continued to be exposed to FA or CAP and mated with normal male mice until the birth of litter 3 (Fig. 1a). Figures 1b and c depict that the litter 2 offspring born by the Pre-Con dams (withdrawn from CAP exposure for 51 days on average) had significantly lower birth weight versus those born by the FA dams, but the litter 3 offspring of the Pre-Con dams (withdrawn from CAP exposure for 134 days on average) had comparable birth weight with the FA counterpart. These data demonstrate that the programming capacity of CAP-exposed dams was reversible, although slowly. In contrast, the litter 2 offspring of the Post-Con dams (exposed to CAP for 55 days on average, given the 21-day-gestation of mice, approximately equaled to a 5-week pre-conceptional exposure) had comparable birth weight with those born by the FA dams, and their litter 3 offspring (exposed to CAP for 127 days on average, approximately equaled to a 15-week pre-conceptional exposure) had significantly lower birth weight versus those born by the FA dams, reaffirming the developmental programming by maternal long-term pre-conceptional exposure to CAP.

### Maternal pre-conceptional exposure to CAP is sufficient to program the offspring’s development and adulthood adiposity

Given its significant effect on the offspring’s birth weight, we next assessed whether maternal pre-conceptional CAP exposure is sufficient to persistently impact the growth trajectory of the litter 1 offspring. Figure 1d and e show that consistent with previous studies [29], maternal exposure to CAP throughout the 7-week pre-conceptional period and the whole gestation and lactation period significantly altered the growth trajectory of male but not female offspring. This was accompanied by increased adiposity in adult male but not female offspring born by CAP dams versus those of FA dams (Fig. 1f-i). The growth trajectory of male offspring born by Pre-Con dams was significantly different from that of FA dams, but comparable with that of CAP dams (Fig. 1d). Specifically, compared to those born by the FA dams, the male offspring of the Pre-Con dams had a “catch-up” growth during their early life, making them have a higher body weight by the postnatal week 2. This difference in body weight was maintained throughout the remaining observation period. We did not observe any significant body weight difference between the offspring born by the Post-Con dams and those born by the FA dams during the lactation period. Therefore, the offspring born by the Post-Con dams were not followed up after weaning.

To further document the programming by maternal pre-conceptional exposure to CAP, the offspring’s body composition in adulthood was assessed. Table 3 reveals that like their CAP counterpart, the adult male offspring of the Pre-Con dams had significantly more adipose mass (epididymal and perirenal fat tissues) than those born by the FA dams. No other significant difference in main organ weight was observed (Table 3). Morphological analysis of the epididymal adipose tissues reveals that compared to those born by the FA dams, the adult male offspring of the Pre-Con and CAP dams had markedly enlarged adipocytes (Fig. 1f and g).

**Table 3:** Organ weights of adult F_1_. All the data were expressed as % of body weight (mean ± SEM). *p < 0.05 versus FA, one-way ANOVA

	Male			Female		
	FA	CAP	Pre-Con	FA	CAP	Pre-Con
Perigonadal fat	1.38 ± 0.14	2.60 ± 0.09*	2.48 ± 0.17*	1.58 ± 0.13	1.92 ± 0.27	1.53 ± 0.25
Perirenal fat	0.52 ± 0.07	1.21 ± 0.06*	1.19 ± 0.17*	0.90 ± 0.06	1.16 ± 0.28	0.96 ± 0.05
Subcutaneous fat	0.68 ± 0.04	0.99 ± 0.03	0.78 ± 0.06	0.99 ± 0.05	1.33 ± 0.16	0.98 ± 0.09
Brown fat	0.31 ± 0.00	0.31 ± 0.00	0.29 ± 0.02	0.21 ± 0.00	0.26 ± 0.05	0.22 ± 0.03
Kidney	1.23 ± 0.01	1.33 ± 0.01	1.31 ± 0.01	1.21 ± 0.02	1.19 ± 0.07	1.28 ± 0.05
Pancreas	0.75 ± 0.00	0.79 ± 0.02	0.75 ± 0.00	0.84 ± 0.00	0.89 ± 0.04	0.96 ± 0.06
Liver	5.12 ± 0.16	4.93 ± 0.05	4.69 ± 0.13	5.02 ± 0.08	4.61 ± 0.11	4.65 ± 0.09
Heart	0.64 ± 0.00	0.64 ± 0.01	0.60 ± 0.01	0.57 ± 0.02	0.50 ± 0.05	0.59 ± 0.03
Lung	0.75 ± 0.01	0.79 ± 0.02	0.75 ± 0.00	1.09 ± 0.08	0.85 ± 0.17	1.01 ± 0.09
Spleen	0.38 ± 0.00	0.32 ± 0.01	0.30 ± 0.01	0.61 ± 0.02	0.52 ± 0.11	0.58 ± 0.03
Testis/Ovary	0.62 ± 0.00	0.66 ± 0.00	0.61 ± 0.05	0.02 ± 0.00	0.02 ± 0.00	0.02 ± 0.00

### Maternal but not paternal transmission of maternal CAP exposure-induced developmental programming of adulthood adiposity

**Fig. 2:**
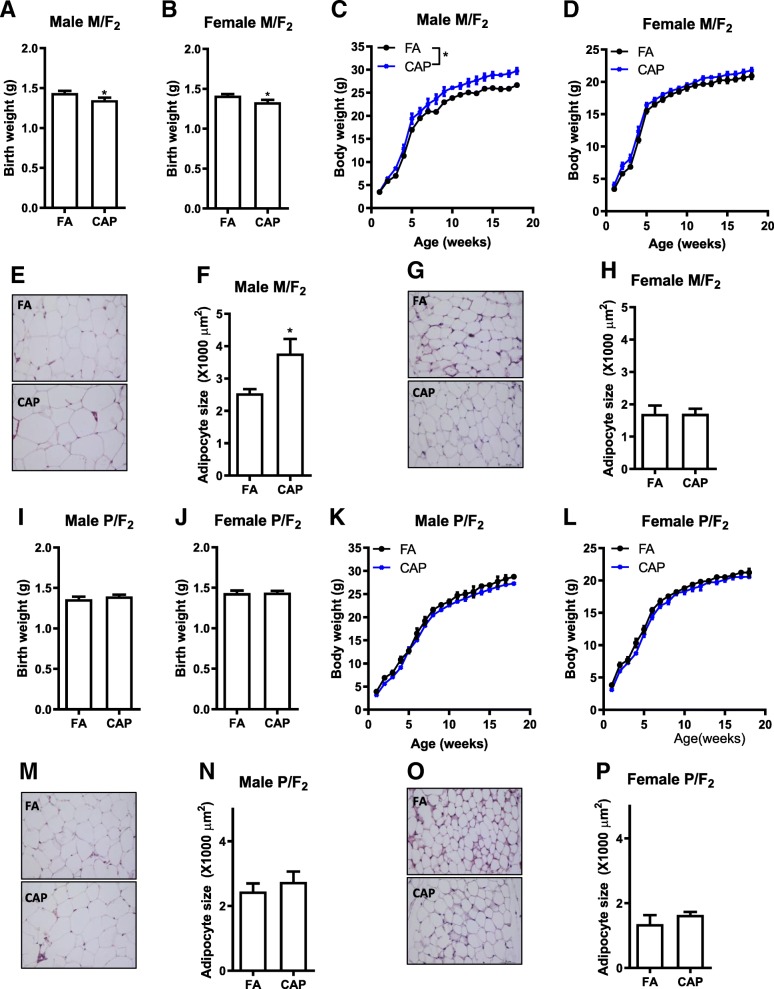
Maternal but not paternal transmission of maternal CAP exposure-induced developmental programming of adulthood adiposity. **a** The birth weight of male F_2_ offspring born by the female F_1_ (the maternal line, M/F_2_). **b** The birth weight of female M/F_2_ offspring. **c** The growth trajectory of male M/F_2_ offspring. **d** The growth trajectory of female M/F_2_ offspring. **e** and **f**. The representative histological (H&E staining) images (**e**) and adipocyte size (**f**) of male M/F_2_ offspring’s perigonadal adipose tissues obtained using ImageJ. **g** and **h**. The representative histological (H&E staining) images (**g**) and adipocyte size (**h**) of female M/F_2_ offspring’s perigonadal adipose tissues obtained using ImageJ. **I**. The birth weight of male F_2_ offspring born by the male F_1_ (the paternal line, P/F_2_). **J**. The birth weight of female P/F_2_ offspring. **K**. The growth trajectory of male P/F_2_ offspring. **L**. The growth trajectory of female P/F_2_ offspring. **M** and **N**. The representative histological (**h** and **e** staining) images (**m**) and adipocyte size (N) of male P/F_2_ offspring’s perigonadal adipose tissues obtained using ImageJ. **O** and **P**. The representative histological (H&E staining) images (**o**) and adipocyte size (**p**) of female P/F_2_ offspring’s perigonadal adipose tissues obtained using ImageJ. *n* = 11–28/group, *p < 0.05 versus FA, student *t* test or two-way ANOVA

**Fig. 3:**
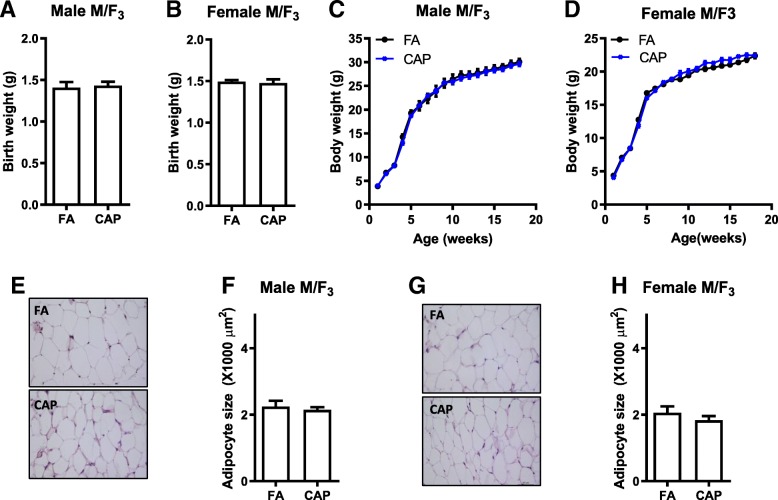
The developmental programming by maternal exposure to CAP is absent in F_3_ offspring. **a** The birth weight of male F_3_ offspring born by the female M/F_2_ (the maternal line. M/F_3_). **b** The birth weight of female M/F_3_ offspring. **c** The growth trajectory of male M/F_3_ offspring. **d** The growth trajectory of female M/F_3_ offspring. **e** and **f**. The representative histological (H&E staining) images (**e**) and adipocyte size (**f**) of male M/F_3_ offspring’s perigonadal adipose tissues obtained using ImageJ. **g** and **h**. The representative histological (H&E staining) images (**g**) and adipocyte size (**h**) of female M/F_3_ offspring’s perigonadal adipose tissues obtained using ImageJ. *n* = 15–19/group, *p < 0.05 versus FA, student *t* test or two-way ANOVA

To determine whether the adverse development in F_1_ offspring can be transmitted into the next generation in the absence of any further CAP exposure, both male and female F_1_ offspring born by the FA and CAP dams were mated with normal C57Bl/6 J mice. Figure 2a and b show that although the growth trajectory of female F_1_ offspring born by the CAP dams was comparable with those of the FA dams (Fig. 1c), their offspring (F_2_ in the maternal line, M/F_2_) had significantly lower birth weight versus the FA counterpart. This was accompanied by increased postnatal weight gain (Fig. 2c), increased adulthood adiposity (Table 4), and enlarged adipocytes (Fig. 3e and f) in the male but not female M/F_2_ of the CAP versus FA dams. In contrast, despite that the male F_1_ offspring of the CAP versus FA dams had significantly different growth trajectory and adulthood adiposity, their offspring (F_2_ in the paternal line, P/F_2_) had comparable birth weight (Fig. 2i and j), similar growth trajectory (Fig. 2k and l), and comparable adulthood adiposity (Table 4 and Fig. 2m-p).

**Table 4:** Organ weights of adult F_2_. All the data were expressed as % of body weight (mean ± SEM). *p < 0.05 versus FA, one-way ANOVA. M/F_2_, F_2_ offspring in the maternal line; P/F_2_, F_2_ offspring in the paternal line

	M/F_2_				P/F_2_			
	Male		Female		Male		Female	
	FA	CAP	FA	CAP	FA	CAP	FA	CAP
Perigonadal fat	1.14 ± 0.02	1.80 ± 0.25*	1.09 ± 0.11	1.30 ± 0.14	0.99 ± 0.14	1.14 ± 0.27	1.08 ± 0.30	1.34 ± 0.17
Perirenal fat	0.45 ± 0.01	0.79 ± 0.18*	0.41 ± 0.04	0.49 ± 0.03	0.37 ± 0.06	0.49 ± 0.06	0.55 ± 0.17	0.45 ± 0.03
Subcutaneous fat	0.57 ± 0.00	0.71 ± 0.07	0.78 ± 0.06	0.79 ± 0.04	0.46 ± 0.03	0.61 ± 0.02	1.00 ± 0.19	0.83 ± 0.04
Brown fat	0.31 ± 0.00	0.37 ± 0.05	0.27 ± 0.00	0.35 ± 0.02	0.27 ± 0.06	0.32 ± 0.00	0.25 ± 0.03	0.30 ± 0.02
Kidney	1.36 ± 0.01	1.32 ± 0.01	1.36 ± 0.03	1.55 ± 0.03	1.38 ± 0.00	1.32 ± 0.01	1.29 ± 0.16	1.48 ± 0.02
Pancreas	0.64 ± 0.01	0.58 ± 0.03	0.61 ± 0.02	0.79 ± 0.03	0.65 ± 0.05	0.71 ± 0.02	0.80 ± 0.05	0.75 ± 0.05
Liver	5.19 ± 0.05	4.84 ± 0.31	5.69 ± 0.16	6.46 ± 0.21	5.50 ± 0.12	5.09 ± 0.02	4.88 ± 0.32	5.24 ± 0.21
Heart	0.54 ± 0.00	0.51 ± 0.02	0.53 ± 0.01	0.58 ± 0.02	0.56 ± 0.00	0.54 ± 0.00	0.55 ± 0.06	0.62 ± 0.02
Lung	0.76 ± 0.01	0.68 ± 0.00	0.81 ± 0.01	0.89 ± 0.00	0.83 ± 0.02	0.73 ± 0.01	0.87 ± 0.02	0.82 ± 0.03
Spleen	0.52 ± 0.03	0.41 ± 0.00	0.42 ± 0.02	0.54 ± 0.03	0.46 ± 0.02	0.36 ± 0.00	0.64 ± 0.02	0.58 ± 0.09
Testis/Ovary	0.79 ± 0.01	0.68 ± 0.02	0.03 ± 0.00	0.04 ± 0.00	0.73 ± 0.00	0.66 ± 0.01	0.02 ± 0.00	0.03 ± 0.00

### The developmental programming by maternal exposure to CAP is absent in F_3_ offspring

Since the abovementioned data show that the adverse development of male offspring due to maternal exposure to CAP was transmitted maternally, the female M/F_2_ were mated with normal male C57Bl/6 J mice to examine how many generations it can be transmitted. Figure 3a -d show that the F_3_ born by the CAP versus FA dams had comparable birth weight and similar growth trajectory. Their body compositions (Table 5) and adipocyte sizes (Fig. 3e-h) were comparable too.

**Table 5:** Organ weights of adult F_3_. These were offspring of female M/F_2_. All the data were expressed as % of body weight (mean ± SEM). *p < 0.05 versus FA, one-way ANOVA

	Male		Female	
	FA	CAP	FA	CAP
Perigonadal fat	2.25 ± 0.09	1.94 ± 0.07	1.41 ± 0.04	1.56 ± 0.05
Perirenal fat	0.98 ± 0.04	0.90 ± 0.02	0.75 ± 0.02	0.77 ± 0.02
Subcutaneous fat	0.96 ± 0.03	0.96 ± 0.02	1.01 ± 0.02	0.99 ± 0.03
Brown fat	0.33 ± 0.00	0.30 ± 0.00	0.31 ± 0.01	0.26 ± 0.01
Kidney	1.28 ± 0.02	1.24 ± 0.01	1.29 ± 0.01	1.25 ± 0.01
Pancreas	0.60 ± 0.01	0.63 ± 0.01	0.68 ± 0.00	0.66 ± 0.00
Liver	4.98 ± 0.04	4.77 ± 0.02	4.75 ± 0.02	4.83 ± 0.03
Heart	0.51 ± 0.01	0.50 ± 0.01	0.55 ± 0.00	0.48 ± 0.00
Lung	0.67 ± 0.00	0.70 ± 0.00	0.84 ± 0.01	0.91 ± 0.02
Spleen	0.31 ± 0.02	0.31 ± 0.01	0.41 ± 0.01	0.40 ± 0.02
Testis/Ovary	0.72 ± 0.00	0.71 ± 0.00	0.04 ± 0.00	0.03 ± 0.00

### The developmental programming of adulthood adiposity by maternal exposure to CAP is accompanied by the decrease in the adipose leptin expression

**Fig. 4:**
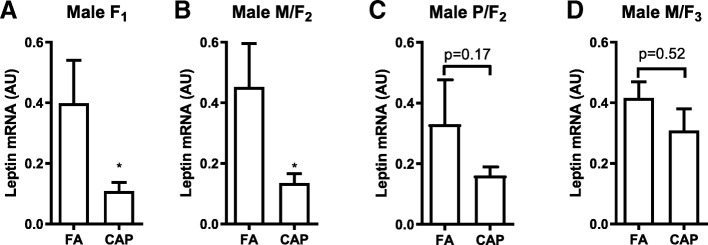
The adipose expression levels of leptin mRNA. Total RNAs were extracted from the epididymal adipose tissues of the indicated male offspring, and the expression levels of leptin mRNA were determined by qPCR. *n* = 12 and 18 (**a**); 18 and 28 (**b**); 11 and 19 (**c**); 15 and 19 (**d**). *p < 0.05 versus FA, student *t* test

We previously demonstrated that the programming of adulthood adiposity by maternal CAP exposure coincides with a decrease in adipose leptin expression [29]. To further document the role of this decreased leptin expression in the developmental programming by maternal CAP exposure, we used qPCR to determine the adipose leptin mRNA expression levels in all three generations of male offspring. Figure 4 reveals that compared to their FA controls, the F_1_ and M/F_2_, but not P/F_2_ nor M/F_3_, offspring of the CAP-exposed dams had significantly decreased expression of leptin mRNA.

## Discussion

Compelling evidence indicates that exposure to environmental stressors during the early life or even the pre-conceptional period may lead to the pathogenesis of various non-communicable diseases, known as the developmental origins (or programming) of health and disease [2]. We previously showed that maternal exposure to CAP throughout the 7-week pre-conceptional period and the whole gestation and lactation period programs the male offspring’s development and adulthood adiposity, raising a new health concern over PM_2.5_ pollution. In the present study, we extended our finding, demonstrating that: (1) maternal pre-conceptional exposure to CAP was sufficient to program the male offspring’s development and adulthood adiposity; and (2) this programming of the male offspring’s development and adulthood adiposity was maternally transmitted cross three generations (from F_0_ to F_2_). These new data not only identify the vulnerable window for the adverse programming of male offspring’s development and adulthood adiposity by maternal exposure to PM_2.5_, but also provide a mechanistic insight into this adverse health effect due to exposure to PM_2.5_.

The composition of PM_2.5_ spatiotemporally varies and may modulate the health effect due to exposure to PM_2.5_. We previously demonstrated that the offspring’s development and adulthood adiposity is persistently effected by maternal exposure to CAP that has a relatively high ratio of Na/Al, which reflects the geographic proximity of the study site (the campus of the University of Maryland, Baltimore) to the ocean [34]. In the present study, we show that the offspring’s development and adulthood adiposity are programmed by maternal exposure to CAP that has relatively high crustal elements including Si, Al, Ti, and Fe, which reflects the undergoing major construction at the exposure site in Shanghai, China [33]. These data collectively suggest that the composition of PM_2.5_ may play a limited role in the developmental programming by maternal exposure to PM_2.5_. This is consistent with the numerous studies performed at different sites but showing similar health effects of exposure to PM_2.5_ [35, 36].

Numerous studies have demonstrated that the timing of exposure to environmental stressor may determine the subsequent developmental programming [2]. As such, identification of the vulnerable window for each adverse programming is of scientific and public health importance. In the present study, a 7-week pre-conceptional period was identified as the vulnerable window for the programming of male offspring’s development and adulthood adiposity by maternal exposure to PM_2.5_. This is evidenced primarily by the data showing that maternal 7-week pre-conceptional exposure to CAP persistently altered male offspring’s development and adulthood adiposity, and all these alterations were comparable with those induced by maternal CAP exposure throughout the 7-week pre-conceptional period and the whole gestation and lactation period (Fig. 1b-e and Table 3). Furthermore, the present study demonstrates that maternal exposure to CAP throughout the whole gestation and lactation period did not impact the offspring’s birth weight and growth trajectory during lactation (Fig. 1). This is consistent with one recent study showing that maternal exposure to CAP during pregnancy did not program the offspring’s growth trajectory [37]. These data collectively rule out the possibility that the gestation and lactation period is vulnerable window for the programming of male offspring’s development and adulthood adiposity by maternal exposure to PM_2.5_.

Notably, the pre-conceptional period has been frequently identified as a vulnerable window for developmental programming by paternal exposure to environmental stressors [38, 39], whereas it has rarely been investigated when identifying the vulnerable window for programming by maternal exposure to environmental stressors. Nonetheless, there are several studies suggesting that maternal pre-conceptional exposure may program offspring’s health. For example, maternal exposure to air pollution before pregnancy was shown to correlate with changes in newborn’s cord blood lymphocyte subpopulations [40], and maternal pre-pregnancy body mass index was shown to modify the association between prenatal traffic-related air pollution exposure and birth weight [41]. Along with these previous studies, the present data strongly suggest that the pre-conceptional period may merit more consideration as a vulnerable window for developmental programming by maternal exposure to environmental stressors.

Furthermore, the identification of the pre-conceptional period as a vulnerable window for maternal exposure to PM_2.5_-induced developmental programming sheds some light on its mechanism, particularly the maternal insult that mediates this adverse programming. As it would benefit the health of both mother and child, to prevent maternal insult is one of the most attractive strategy to stop maternal exposure-induced adverse programming. The sufficiency of pre- but not post-conceptional maternal exposure to CAP programing offspring’s development and adulthood adiposity calls special attention to the role of maternal gametic insult in this adverse programming, in contrast to the most frequently studied maternal somatic insult such as placenta dysfunction. The implication of maternal gametic insult is also strongly supported by the maternal transmission of this adverse programming into the F_2_ generation (Fig. 2), which is another important finding in the present study. This demonstration of three-generational effects obviously raises more public health concerns over the adverse programming by maternal exposure to PM_2.5_. Given the absence of adverse development in F_3_ generation (Fig. 3), it is however not a true transgenerational inheritance, which requires at least four-generational maternal transmission or three-generational paternal transmission. Nonetheless, as both F_1_ and F_2_ offspring were not subject to any further CAP exposure, this three-generational maternal transmission clearly demonstrates that maternal gametic insult is involved in this adverse developmental programming.

Low birth weight correlates with a variety of short- and long-term health problems. There is a rapidly increasing body of literature showing that maternal exposure to PM_2.5_ correlates with low birth weight [36]. Interestingly, the cross-generational investigation reveals a clear coincidence of low birth weight, accelerated postnatal growth, and increased adulthood adiposity. This is consistent with numerous studies showing that low birth weight is a risk factor for various cardiometabolic diseases including obesity [42]. These collectively suggest a causal role of low birth weight in the adverse programming of growth trajectory and adulthood adiposity by maternal exposure to PM_2.5_, making the former a valuable indicator to predict cross-generational effect due to exposure to PM_2.5_. However, it should be noted that there are conflictive studies too. For example, one recent study revealed that maternal exposure to CAP during pregnancy resulted in offspring’s low birth weight but not change in growth trajectory [37]. Given the rapidly increasing evidence that maternal exposure to PM_2.5_ correlates with low birth weight, further study is urgently needed to verify its role in developmental programming due to exposure to PM_2.5_.

An additional important finding in the present study is that the male offspring only manifests maternal CAP exposure-induced accelerated postnatal weight gain and increased adulthood adiposity, whereas the female offspring only transmits these traits into the next generation. This segregation of the programming capacity and the programmed traits is impressive, suggesting that the accelerated postnatal growth and increased adulthood adiposity are unlikely components of a self-sustaining loop that is essential for a true transgenerational inheritance. It further supports the implication of maternal gametic insult in the transmission of this adverse developmental programming.

## Conclusion

The present study demonstrates that maternal pre-conceptional exposure to PM_2.5_ adversely programs male offspring’s development and adulthood adiposity, which is maternally transmitted cross three generations. These data not only call special attention to the protection of women from exposure to PM_2.5_, but also lay some foundation for the development of intervention to prevent the adverse developmental programming by maternal exposure to PM_2.5_.

## Data Availability

The datasets used and/or analysed during the current study are available from the corresponding author on reasonable request.

## **Abbreviations**

ANOVA: Analysis of variance
BAT: Brown adipose tissue
CAP: Concentrated ambient PM_2.5_
DEP: Diesel exhaust PM_2.5_
DOHaD: Developmental origins of health and disease
FA: Filtered air
PM_2.5_: Ambient fine particulate matter
VACES: Versatile aerosol concentration enrichment system
